# EWS/FLI utilizes NKX2-2 to repress mesenchymal features of Ewing sarcoma

**DOI:** 10.18632/genesandcancer.57

**Published:** 2015-03

**Authors:** John Fadul, Russell Bell, Laura M. Hoffman, Mary C. Beckerle, Michael E. Engel, Stephen L. Lessnick

**Affiliations:** ^1^ Huntsman Cancer Institute, School of Medicine, University of Utah, Salt Lake City, Utah, USA; ^2^ Department of Oncological Sciences, School of Medicine, University of Utah, Salt Lake City, Utah, USA; ^3^ Department of Biology, School of Medicine, University of Utah, Salt Lake City, Utah, USA; ^4^ Center for Children's Cancer Research, School of Medicine, University of Utah, Salt Lake City, Utah, USA; ^5^ Division of Pediatric Hematology and Oncology, School of Medicine, University of Utah, Salt Lake City, Utah, USA

**Keywords:** Ewing sarcoma, NKX2-2, EWS/FLI, mesenchymal, adhesion

## Abstract

In Ewing sarcoma, NKX2-2 is a critical activated target of the oncogenic transcription factor EWS/FLI that is required for transformation. However, its biological function in this malignancy is unknown. Here we provide evidence that NKX2-2 mediates the EWS/FLI-controlled block of mesenchymal features. Transcriptome-wide RNA sequencing revealed that NKX2-2 represses cell adhesion and extracellular matrix organization genes. NKX2-2-depleted cells form more focal adhesions and organized actin stress fibers, and spread over a wider area—hallmarks of mesenchymally derived cells. Furthermore, NKX2-2 represses the actin-stabilizing protein zyxin, suggesting that these morphological changes are attributable to zyxin de-repression. In addition, NKX2-2-knockdown cells display marked increases in migration and substrate adhesion. However, only part of the EWS/FLI phenotype is NKX2-2-dependent; consequently, NKX2-2 is insufficient to rescue EWS/FLI repression of mesenchymalization. Strikingly, we found that EWS/FLI-and NKX22-repressed genes are activated by ZEB2, which was previously shown to block Ewing sarcoma epithelialization. Together, these data support an emerging theme wherein Ewing sarcoma cells highly express transcription factors that maintain an undifferentiated state. Importantly, co-opting epithelial and mesenchymal traits by Ewing sarcoma cells may explain how the primary tumor grows rapidly while also “passively” metastasizing, without the need for transitions toward differentiated states, as in carcinomas.

## INTRODUCTION

Translocation fusions between the various FET (FUS-EWS-TAF15) and ETS (E twenty-six) genes are the causative agent of Ewing sarcoma [1, 2]. Of these, the t(11;22)(q24;q12) lesion that encodes the oncogenic fusion protein EWS/FLI is by far the most common, being present in 85% of all Ewing sarcoma tumors [2]. The fusion of the DNA-binding domain of *FLI1* with the strong transactivation domain of *EWSR1* yields an aberrant transcription factor. Although genomic sequencing of tumor samples revealed occasional loss of *STAG2* and *CDKN2A*, as well as mutations in *TP53*, this pediatric malignancy is largely genomically stable, suggesting that transcriptional dysregulation by EWS/FLI is the primary oncogenic driver [3-5]. We have previously shown that EWS/FLI is able to directly activate genes by binding GGAA microsatellite-rich promoters, while others demonstrated its binding with the transcriptional co-activator CBP/p300, or phosphorylation-dependent interaction with the C-terminal domain of RNA PolII itself [6-12]. Recently, we found that EWS/FLI directly represses genes by binding an as yet uncharacterized consensus sequence and recruiting histone deacetylases (HDACs) and lysine-specific demethylase 1 (LSD1) [13, 14]. We realize, however, the importance of a “second wave” of gene regulation that occurs when EWS/FLI modulates the expression of other transcription factors; for instance, we previously identified the homeobox transcription factor NKX2-2 as a critical upregulated target of EWS/FLI [15].

NKX2-2 has well-characterized roles in normal development. Deletion of its fly ortholog *vnd* (ventral nervous system defective) results to a complete loss of the ventral region of the central nervous system (CNS) and embryonic lethality [16, 17]. In the mammalian CNS, NKX2-2 specifies V3 interneurons, controls oligodendrocyte differentiation, and guides floor plate development [18-22]. It is critical in the fate specification of enteroendocrine cells in the small intestine and insulin-producing β-islet cells in the pancreas, where several of its transcriptional targets have been identified and characterized [23-32]. Mice homozygous for a null *Nkx2.2* mutation die postnatally due to a complete loss of β-islet cells and severe hyperglycemia [24]. Mechanistically, NKX2-2 has been shown to bind Gro/TLE co-repressors, which can then recruit HDACs, to effect transcriptional repression [33, 34]. Conversely, a C-terminal transactivation domain is unraveled when the neighboring NK2-specific domain is deleted, suggesting that NKX2-2 may also function as a transcriptional activator in some contexts [35, 36].

In Ewing sarcoma, we demonstrated that NKX22 is necessary for the maintenance of transformation *in vitro* and *in vivo* [15, 37]. Transcriptional profiling of NKX2-2 and EWS/FLI using microarrays revealed that they share a repressed geneset. Furthermore, Ewing sarcoma cells overexpressing AES (amino enhancer of split), a dominant-negative Gro/TLE protein, or treated with the HDAC inhibitor vorinostat display diminished transformation, consistent with NKX2-2 recruitment of Gro/TLE co-repressors and HDACs. Strikingly, vorinostat treatment completely reverses the transcriptional profile of NKX2-2, suggesting that NKX2-2 represses genes via modification of histone acetyl-lysine marks in Ewing sarcoma cells. Further analysis using domain deletion mutants demonstrated that DNA binding, transcriptional repression, and inhibition of transcriptional activation are necessary for the transformed phenotype [37]. Importantly, NKX2-2 was shown to be a sensitive and specific diagnostic marker of Ewing sarcoma versus other look-alike small round blue-cell tumors (SRBCTs), both at the RNA and protein levels [15, 38, 39].

In this current work, we perform unbiased transcriptional profiling of NKX2-2 using RNA-seq, which revealed that NKX2-2 downregulates genes critical for cell adhesion. This led to the discovery that EWS/FLI represses cell adhesion and other mesenchymal characteristics through upregulation of NKX2-2.

## RESULTS

**Figure 1:**
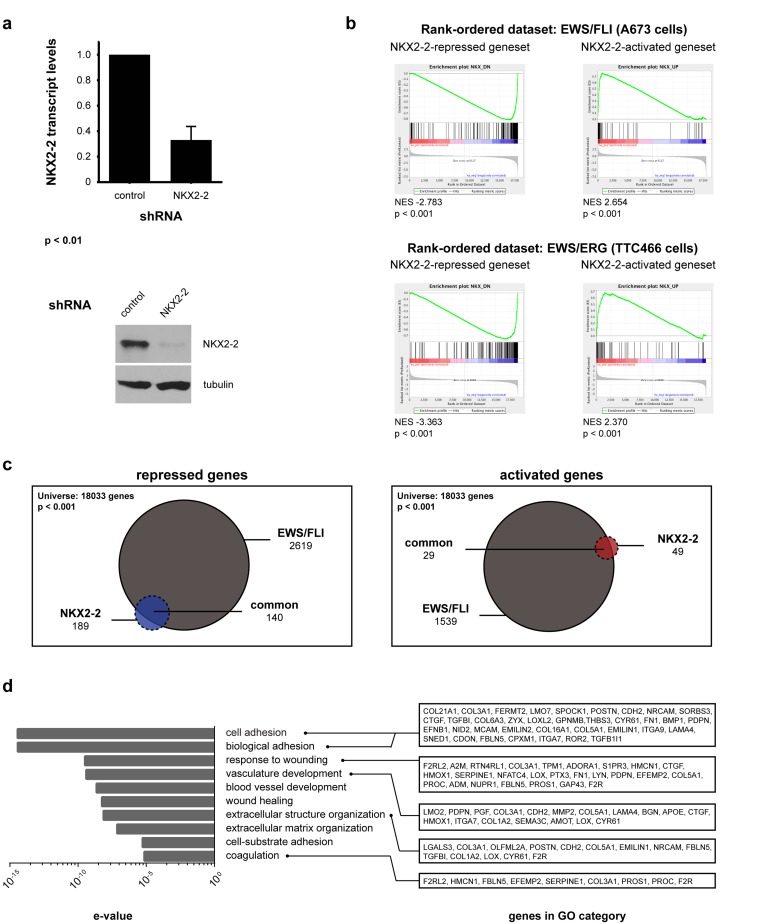
RNA-seq transcriptional profiling of NKX2-2 in the Ewing sarcoma cell line A673 (a) Expression of NKX2-2 is knocked down using retrovirally delivered shRNA specific to the 3′-UTR, as shown by qRT-PCR for NKX2-2 transcript levels (normalized to GAPDH levels) and western blot for protein levels (tubulin, loading control). (b) GSEA shows that NKX2-2-activated or repressed genes are enriched in the EWS/FLI-upregulated or downregulated dataset, respectively, from A673 cells. The same is true for the EWS/ERGdataset from TTC466 cells. (c) Venn diagram analysis of the NKX2-2 and EWS/FLI repressed and activated genesets. The 140 common repressed genes and 29 common activated genes are highlighted in Table S1. (d) DAVID gene ontology analysis reveals that the NKX2-2repressed geneset is enriched for cell adhesion genes. Genes from key GO terms are listed on the right.

We previously reported that NKX2-2 is a critical upregulated target of EWS/FLI, and that it is necessary for the maintenance of transformation. Since it is a member of the NK family of homeobox-binding transcription factors and has been demonstrated to control gene expression in many developmental contexts, we reasoned that NKX2-2 depletion and transcriptional profiling would best reveal its function in Ewing sarcoma. We had previously determined the NKX2-2 transcriptional profile using microarrays [37]; to reduce bias, capture the full transcriptional profile, and enable direct comparison with our recent EWS/FLI dataset [14] we elected to perform RNA-seq. To this end we knocked down NKX2-2 expression in the Ewing sarcoma cell line A673 using retrovirally delivered shRNA (Fig. 1a). We then performed Illumina deep sequencing of mRNA isolated from these derivative polyclonal lines. Differential gene expression was determined with USeq tools. Using cutoff parameters of 1.5-fold change and FDR ≥ 50, we found 189 genes repressed and 49 genes activated by NKX2-2 (Table S1, Fig. 1c). The top regulated genes and heat maps are shown in Fig. S1a. We proceeded to validate select repressed and activated targets by qRT-PCR (Fig. S1b).

Gene set enrichment analyses (GSEA) revealed that NKX2-2-repressed genes are enriched in the EWS/FLI-repressed dataset, and NKX2-2-activated genes are enriched in the EWS/FLI-activated dataset (Fig. 1b). We also show that the NKX2-2-and EWS/FLI-repressed genesets, as well as the NKX2-2-and EWS/FLI-activated genesets, significantly overlap, consistent with GSEA data (Fig. 1c). This confirms microarray data we previously published and suggests that modulation of NKX2-2, and indeed any other transcription factor, can be utilized by EWS/FLI to amplify its transcriptional effect [37]. In addition, the NKX2-2-repressed and activated genesets are also appropriately well-represented in the dataset of EWS/ERG, a variant translocation harbored by TTC466 cells, suggesting that regulation of genes through NKX22 is translocation type-independent (Fig. 1b, Fig. S1c) [14]. Further GSEA analyses indicated that the NKX22-regulated genesets are not enriched in transcriptional profiles of rhabdomyosarcomas driven by PAX-FKHR fusions (data not shown) [40]. Thus, the NKX2-2 signature is specific for Ewing sarcoma.

**Figure 2:**
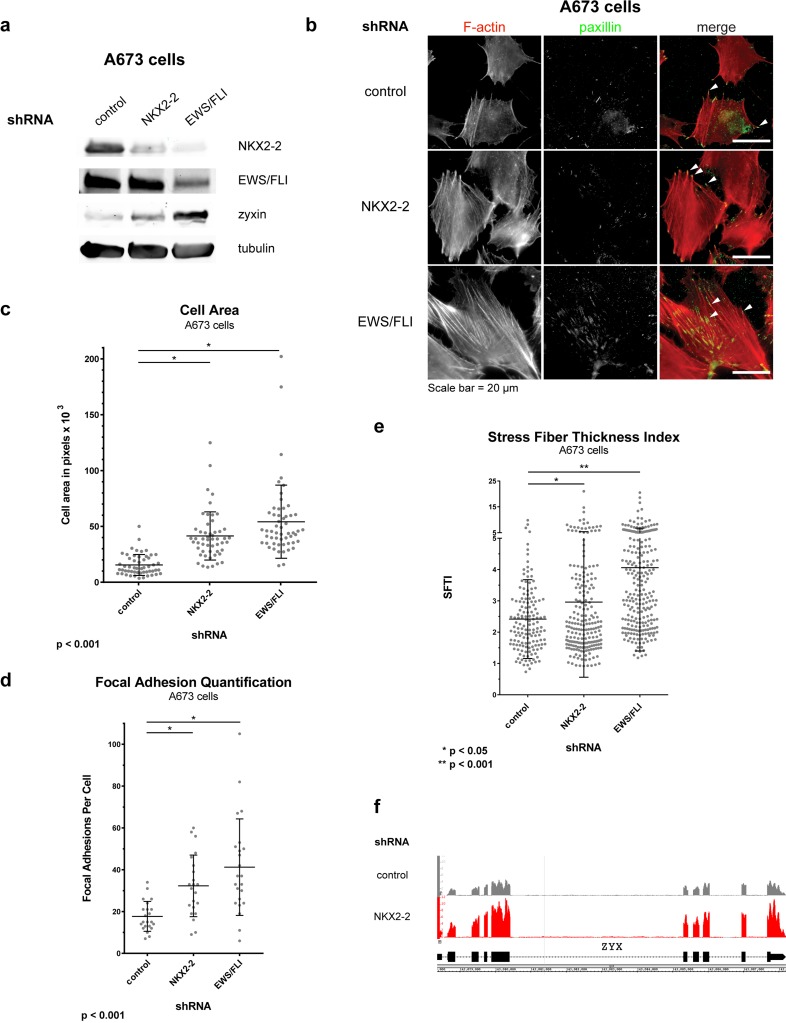
NKX2-2 impairs the capacity of A673 Ewing sarcoma cells to spread on the substrate and to form actin stress fibers and focal adhesions (a) Western blots showing EWS/FLI, NKX2.2, zyxin, and tubulin protein levels for each condition. (b) NKX2-2 or EWS/FLI knockdown significantly increases cell area and organizes the actin cytoskeleton compared to control knockdown. At least 10 fields were taken for each condition. White arrowheads indicate paxillin-positive focal adhesions in the merged channel. (c-e) Quantification of (c) cell area, (d) number of focal adhesions, and (e) stress fiber thickness index in A673 cells. Scatter dot plot indicates individual measurements; mean ± SD is shown over the dot plots. Experiments were done in triplicate at least thrice; shown here is a single representative experiment. (f) Zyxin is de-repressed upon NKX2-2 knockdown. Shown is an Integrated Genome Browser snapshot of the *ZYX* locus (chr7:143,078,360-143,088,206; hg19) from the NKX2-2 RNA-seq differential expression analysis; there are significantly more reads in the NKX2-2 knockdown track than in control knockdown track.

Gene ontology (GO) analysis is widely used to determine the biological functions and cell processes for which a gene is important. We used DAVID functional annotation clustering for GO analysis, and found that NKX2-2 repressed genes important for cell adhesion, wound healing, and extracellular matrix (ECM) organization, among other GO terms identified (Fig. 1d). This suggested that NKX2-2 may contribute to EWS/FLI-mediated impairment of mesenchymal features of Ewing sarcoma cells, which was previously reported [41, 42]. Indeed, NKX2-2 knockdown in A673 cells (Fig. 2a) resulted in spreading over a significantly wider area on a fibronectin substrate compared to cells expressing a control shRNA, phenocopying loss of EWS/FLI (Fig. 2b, 2c; Fig. S2) [41]. Furthermore, while in control knockdown cells there is a diffuse signal of phalloidin staining, indicating short fragments of filamentous actin, upon NKX2-2 or EWS/FLI depletion actin organizes into long, thick stress fibers (Fig. 2b). Using a previously reported metric called stress fiber thickness index (SFTI), we determined that these increases in actin organization are indeed significant (Fig. 2e) [43, 44].

Cell spreading is a prominent feature of mesenchymally derived cells and occurs due to increased substrate adhesion. Adhesion is a complex process involving binding of various integrins to their cognate motifs in ECM proteins, signaling through adaptor proteins, and consequent organization of attached cytoskeletal elements. As predicted, we observed that significantly more focal adhesions (FA) form upon NKX2-2 or EWS/FLI knockdown, as shown by immunofluorescence staining for paxillin, one of the myriad proteins that populate FAs (Fig. 2b, 2d). Importantly, with all morphological phenotypes observed, the effect with NKX2-2 loss is less dramatic than EWS/FLI loss. This is not surprising because the NKX22-repressed geneset, while significantly overlapping with that of EWS/FLI, is only a small subset (Fig. 1c). Presumably EWS/FLI activates or represses the expression of other genes that control different aspects of formation of FAs and actin stress fibers, leading to increases in cell area.

An EWS/FLI-repressed gene that has been shown to have an effect on mesenchymal traits is zyxin. This protein is a FA component and has been demonstrated to aid in the stabilization of actin stress fibers [45]. The de-repression of *ZYX* might partially explain how NKX2-2 or EWS/FLI knockdown allows Ewing sarcoma cells to manifest mesenchymal characteristics. Predictably, when we knock down NKX2-2, zyxin is upregulated, as shown both by western blot (Fig. 2a) or by RNA-seq reads mapping to the *ZYX* locus (Fig. 2f).

**Figure 3:**
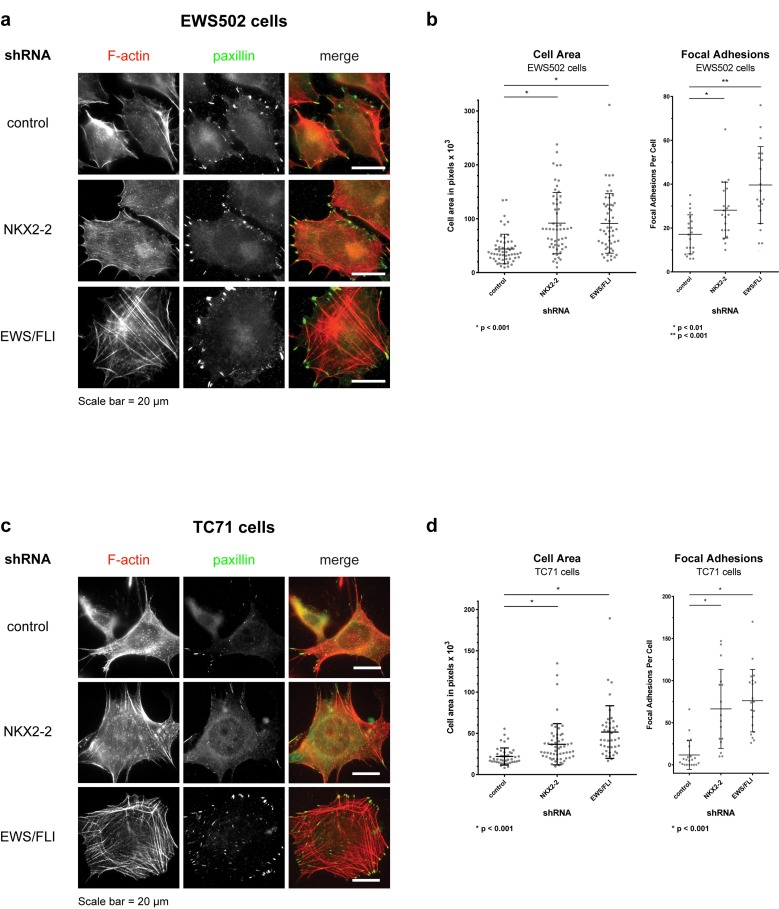
NKX2-2 represses mesenchymal features in two other Ewing sarcoma cell lines (a,c) NKX2-2 or EWS/FLI deficiency significantly increases cell area and organizes the actin cytoskeleton compared to control in (a) EWS502 and (c) TC71 cells. At least 10 fields were taken for each condition. (b,d) Quantification of cell area and number of focal adhesions in (b) EWS502 and (d) TC71 cells.

Notably, these morphological changes were recapitulated in EWS502 and TC71, two other Ewing sarcoma lines with EWS/FLI translocations, demonstrating that this phenomenon is not cell line-specific (Fig 3a,c; Fig. S3, S4). Specifically, cell area and number of focal adhesions increase when NKX2-2 or EWS/FLI is knocked down in these cells (Fig 3b, 3d). In addition, while F-actin signals remain diffuse in EWS502 and TC71 control knockdown cells, actin stress fibers form upon NKX22 or EWS/FLI depletion in either cell line, with thicker and more pronounced fibers in the EWS/FLI knockdown condition (Fig. 3a, 3c). Though less efficient than in A673 cells, we were able to achieve appreciable knockdown of NKX2-2 in EWS502 cells as shown by protein levels, and in TC71 cells as shown by both mRNA and protein levels (Fig. S5). In addition, in these two cell lines zyxin is de-repressed slightly upon NKX2-2 depletion, and markedly upon EWS/FLI depletion, mirroring our initial findings in A673 cells (Fig. S5). Taken together, these data suggest that the underlying transcriptional networks leading to cell adhesion are conserved in all three cell lines used, and may be representative of Ewing sarcoma tumors.

**Figure 4:**
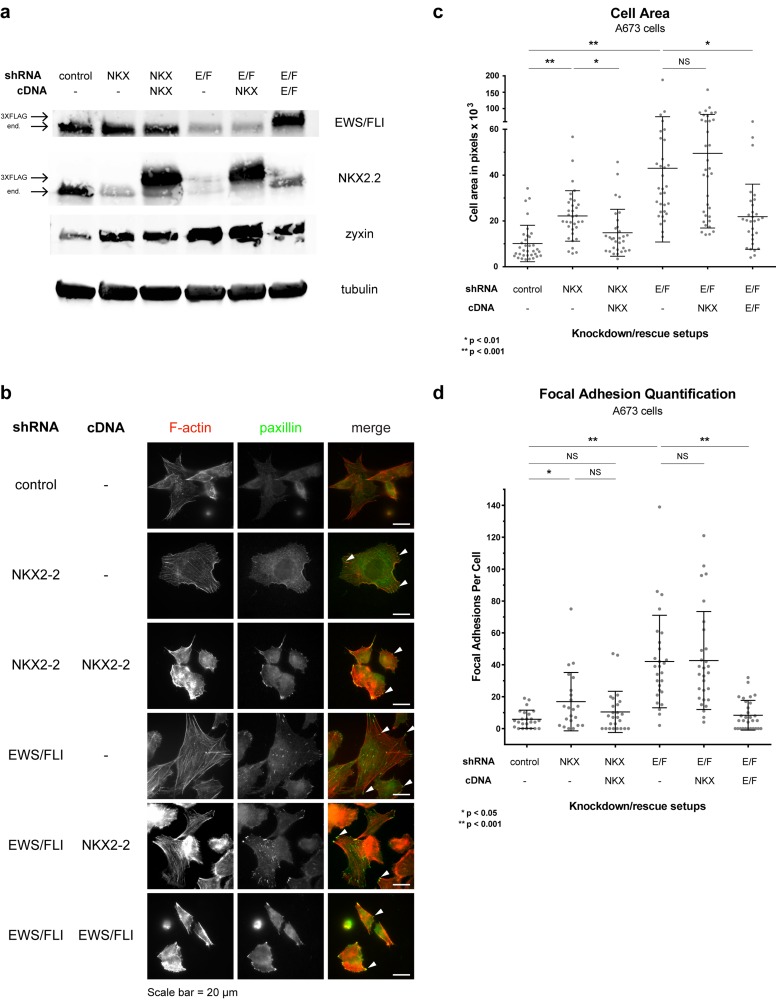
NKX2-2 is necessary but not sufficient for EWS/FLI-mediated repression of mesenchymal features of Ewing sarcoma cells (a) Western blots showing EWS/FLI, NKX2.2, zyxin, and tubulin protein levels for all knockdown-rescue conditions. The bands for endogenous and exogenous 3XFLAG-tagged versions of EWS/FLI and NKX2.2 are indicated by arrows on the left. (b) Immunofluorescence experiments on re-expression studies. At least 10 fields were taken for each condition. White arrowheads indicate paxillin-positive focal adhesions in the merged channel. (c) Quantification of cell area in (b). (d) Quantification of focal adhesions in (b).

To attribute increases in cell area and number of focal adhesions specifically to depletion of NKX2-2 either by an NKX2-2-specific shRNA or by knocking down EWS/FLI, we performed rescue experiments in A673 cells (Fig. 4a). The increase in cell area due to NKX2-2 knockdown was partially rescued by the RNAi-resistant cDNA construct of NKX2-2, confirming that our shRNA has few off-target effects (Fig. 4b, 4c; Fig. S6). There is also partial rescue of the increase in number of FAs (Fig. 4d). As had been shown before, EWS/FLI re-expression completely rescues EWS/FLI knockdown [41]. However, NKX2-2 re-expression upon EWS/FLI knockdown does not rescue phenotype, demonstrating that NKX2-2 is not sufficient to mediate the full complement of EWS/FLI repression of mesenchymal features (Fig. 4b-4d). This is not unexpected because we already observed that NKX2-2 confers only a partial phenotype in three cell lines. Moreover, it also supports our previous data showing that NKX2-2 is necessary but not sufficient for the transformation of Ewing sarcoma cells [37].

**Figure 5:**
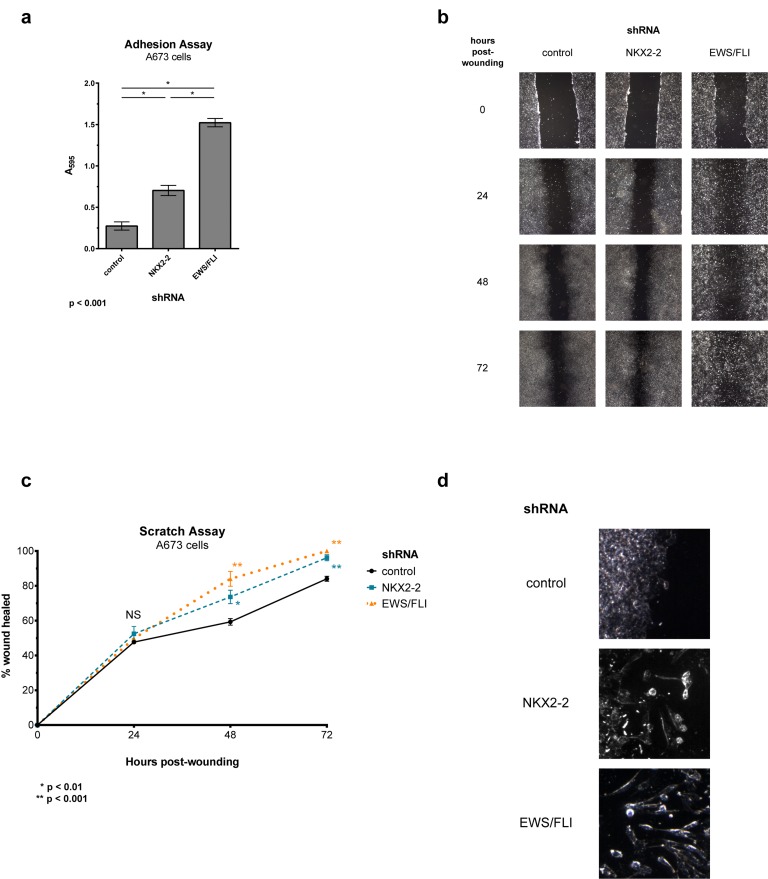
NKX2-2 inhibits cell adhesion and migration (a) NKX2-2-or EWS/FLI-knockdown cells adhere more efficiently to the substrate than control cells. (b) NKX2-2 deficiency increases migration capacity in a scratch assay. Experiments were done at least thrice in A673 cells line using nine replicate fields per treatment. Shown is a single representative experiment. (c) Quantification of (b). Migration is shown as percentage of wound healed compared to the original area of scratch. Error bars indicate SEM. (d) Closer inspection of the monolayer edge reveals cells with mesenchymal features in NKX2-2-or EWS/FLI-knockdown cells, but not in control cells.

Since Ewing sarcoma cells form more focal adhesions upon depletion of NKX2-2, we reasoned that they also adhere more effectively to the substrate. Indeed we found that more NKX2-2-knockdown cells adhere after a 2-h incubation than control-knockdown cells, again phenocopying EWS/FLI knockdown, albeit to a less degree (Fig. 5a) [41].

A hallmark feature of mesenchymal cells is their pronounced capacity to migrate. In addition to impairing cell spreading and adhesion, it was previously demonstrated that EWS/FLI impedes the migration of Ewing sarcoma cells [41]. We therefore asked whether NKX2-2 also contributes to this phenotype. Indeed, in a monolayer scratch assay, depletion of NKX2-2 allows cells to more rapidly heal the wound than control-knockdown cells, but more slowly than EWS/FLI-knockdown cells (Fig. 5b, 5c). Importantly, this is not due to disparities in cell proliferation, as we have previously shown that these shRNAs do not profoundly impact growth in tissue culture [15, 37]. Upon closer examination of the monolayer edge we observed that NKX2-2-and EWS/FLI-knockdown cells migrate actively by elongating and spreading into the wound a feature that is absent in control knockdown cells, where wound healing seems to occur mainly by proliferation (Fig. 5d).

**Figure 6:**
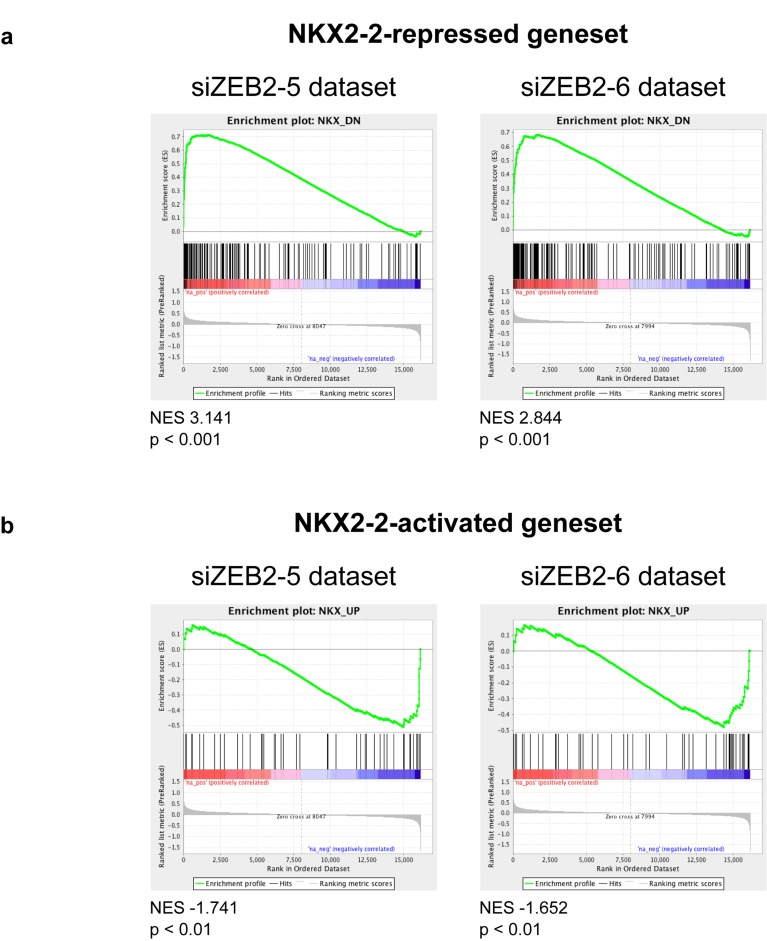
The NKX2-2 and ZEB2 transcriptional profiles are inversely correlated (a) The NKX2-2 repressed geneset is enriched in the ZEB2 upregulated dataset, using two independent siRNAs against ZEB2. (b) The NKX2-2 activated geneset is enriched in the ZEB2 downregulated dataset.

Recently, we showed that the transcription factor and mesenchymal differentiation gene ZEB2 was highly expressed in both mesenchymal stem cells and Ewing sarcoma cell lines. Knockdown of ZEB2 in Ewing sarcoma cells induced morphological changes, such as a pronounced actin ring around the cell periphery, a cobblestone cell shape, and hampered cell migration in a scratch assay. These are in stark contrast to the EWS/FLI-depleted phenotype and strongly suggested that ZEB2 repressed epithelial features of Ewing sarcoma [46]. We reasoned that, since NKX2-2 loss phenocopied EWS/FLI loss, the NKX2-2 and ZEB2 transcriptional profiles must be inversely correlated, and this is indeed the case (Fig. 6). Using datasets previously generated with two independent siRNAs against ZEB2 in A673 cells [46], we found that the NKX2-2-repressed geneset, which contains the genes important for cell adhesion and ECM organization, is enriched in the ZEB2-upregulated dataset (Fig. 6a). Conversely, the NKX2-2-activated geneset is enriched in the ZEB2-downregulated dataset, but with lower normalized enrichment scores (Fig. 6b). Thus, the ZEB2 transcriptional profile which represents an “epithelialized” Ewing sarcoma, and the NKX2-2 transcriptional profile which represents a “mesenchymalized” Ewing sarcoma, antagonize each other. We therefore propose that EWS/FLI, through upregulation of NKX2-2, and ZEB2 act to repress differentiation in opposite directions along the epithelial-mesenchymal spectrum; together, they maintain Ewing sarcoma cells in a partially differentiated state.

## DISCUSSION

**Figure 7:**
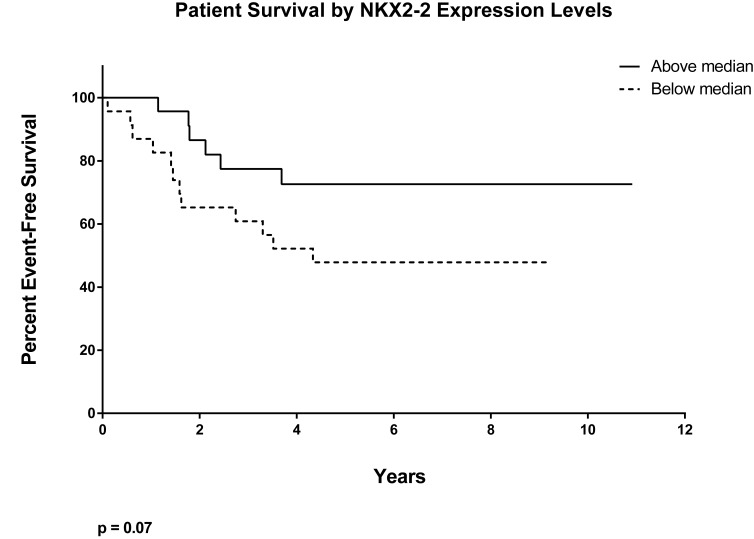
Low expression of NKX2-2 may predict poor patient outcome Kaplan-Meier curve showing patient survival data for above-and below-median NKX2-2 expression (p = 0.07).

NKX2-2 is one of the most highly upregulated genes by EWS/FLI [15]. It has been shown to have diagnostic significance, and may predict patient outcome (Fig. 7), but has thus far been used in the literature only as a marker for EWS/FLI transcriptional activation [47-50]. Our current work fills in this gap and identifies the importance of NKX2-2 as a tool of EWS/FLI in repressing salient mesenchymal characteristics of Ewing sarcoma cells, first displayed by unbiased transcriptional profiling. In summary, we show here that NKX2-2 assists in inhibiting cell spreading and formation of focal adhesions, as well as adhesion and migration in functional assays, and consequently mediates a block in mesenchymal differentiation.

Perhaps one of the most interesting findings of this current study is that NKX2-2 effects only a partial phenotype compared to EWS/FLI-this is true for all characteristics assayed. This underscores the enormity of the role of EWS/FLI, modulating a multitude of genes that each contribute partially to an attribute of tumorigenesis and/or metastasis. Because of the particularly low frequency of other genetic alterations, EWS/FLI acts as a “master regulator” of tumor growth and spread. Consequently, it was not surprising that NKX2-2 re-expression was insufficient for the EWS/FLI-mediated block of mesenchymalization. That several EWS/FLI transcriptional targets, such as NR0B1, GLI1, and indeed NKX2-2 itself, determined to be necessary for transformation are insufficient implies that such a genetic relationship could also be true for other phenotypes, such as cell adhesion and migration [15, 51, 52].

Recently, it was shown that zyxin and α5 integrin are repressed targets of EWS/FLI [42]. Specifically, when EWS/FLI is knocked down, expression of these two proteins go up, concomitant with the display of increased actin stress fiber organization, cell area, and number of FAs. Furthermore, when they are re-expressed individually without EWS/FLI depletion, the same mesenchymal traits manifest. Interestingly, zyxin and α5 integrin double re-expression is sufficient to capture the full EWS/FLI phenotype. Importantly, double re-expression also reduces colony growth in soft agar, suggesting that these two genes somehow contribute to transformation, while simultaneously shortening the latency of metastatic lesions in an intratibial xenograft model [42]. That enforced expression of zyxin and α5 integrin in concert is sufficient to counter the repression of mesenchymal features by EWS/FLI explains the part of the phenotype that cannot be attributed to NKX2-2. However, zyxin and α5 integrin double re-expression does not cause the full loss of transformation that depletion of NKX2-2 does [37], suggesting that there are additional, uncharacterized transcriptional targets of NKX2-2 that contribute to other arms of tumorigenesis.

During normal development, the differentiation state of a cell dictates many aspects of its life: proliferative capacity, interaction with other cells and with its microenvironment, and function. This also holds true for cells in a malignant context. While the cell-of-origin has been identified for the majority of carcinomas, this remains a challenge for Ewing sarcoma, nearly a century after it was first reported in the literature [53]. Neural crest cells and mesenchymal stem cells (MSCs) have had the most evidence for being the cell-of-origin for this disease, as reviewed previously [54]. In summary, Ewing sarcoma has been shown to express neural cell surface antigens; however, it has also been suggested that EWS/FLI has transcriptional control over neurogenesis and that this has little to do with the permissive cell type in which the translocation occurs [54-60]. On the other hand, when EWS/FLI is depleted from Ewing sarcoma cells, a transcriptional profile that closely mimics that of MSCs is adopted [50]. Enforced EWS/FLI expression in mesenchymal cells populating the developing mouse limb bud yields a SRBCT, but only upon p53 deletion [61]. However, while human MSCs expressing EWS/FLI displayed a transcriptional profile resembling that of Ewing sarcoma, injection into immunocompromised mice did not result in any viable tumors [49]. It has also been suggested that these two theories may not be mutually exclusive [54, 62, 63].

Although our work does not directly address the cell-of-origin question, it is consistent with an emerging theme whereby highly expressed proteins in Ewing sarcoma inhibits terminal differentiation. As was proposed before, the translocation perhaps occurs in a progenitor cell that is even less differentiated than MSCs [46]; EWS/FLI then upregulates NKX2-2 to repress mesenchymalization. ZEB2 and the interaction of endogenous EWS and RE1-silencing transcription factor (REST) then repress epithelialization and neuralization, respectively [46, 64]. Interestingly, using a dataset in a recent study of Ewing sarcoma patients from the Children's Oncology Group, we found that below-median expression of NKX2-2 correlates with decreased event-free survival (Fig. 7, p = 0.07) [65]. Volchenboum and colleagues further identified GO terms that are enriched in non-survivors versus survivors: migration, motility, and adhesion the very cell processes that EWS/FLI and NKX2-2 repress [65]. Collectively, our findings suggest that, while NKX2-2 is required for Ewing sarcomagenesis [37], there is a gradation of NKX2.2 levels in patient tumors that may contribute to outcome. Furthermore, high expression of ZEB2 also correlates with decreased survival [46]. Presumably, tumor cells in patients with low NKX2.2 levels and/or high ZEB2 levels are further along mesenchymal differentiation and may exhibit pronounced migratory characteristics, allowing them to metastasize to distal sites with enhanced capacity.

## MATERIALS AND METHODS

### Cell culture and knockdown-rescue infections

The Ewing sarcoma cell lines A673, EWS502, and TC71 were cultured, and retroviruses packaged in HEK293-EBNA cells, using standard techniques previously described [55, 56]. For RNA interference experiments, cells were infected with pMKO1.P retrovirus harboring shRNA constructs against luciferase (control), NKX2-2, or EWS/FLI. For cDNA rescue experiments, cells were infected with pMSCV-hygro retrovirus harboring cDNA for 3Xflag::NKX2-2 or 3Xflag::EWS/FLI, or an empty construct. The cloning strategy for these constructs and methods for retroviral infection and polyclonal selection are detailed elsewhere [15, 56].

### Antibodies and western blotting

Standard western blot techniques were employed, using the following antibodies: goat α-NKX2.2 (Santa Cruz sc-15015, 1:200), rabbit α-FLI1 (Abcam ab15289, 1:1000), rabbit α-zyxin (kind gift from Mary Beckerle, 1:10000), mouse α-tubulin (Calbiochem CP06, 1:1000). HRP-conjugated secondary antibodies were used at 1:1000 dilution: donkey α-goat IgG (Santa Cruz sc-2020), sheep α-rabbit IgG (GE Healthcare NA934V), sheep α-mouse IgG (GE Healthcare NA931V). For western blots visualized using the Li-Cor Infrared Imaging System, their proprietary fluorophore-conjugated secondary antibodies were used at 1:2000 dilution: IRDye 680CW goat α-mouse IgG, IRDye 680CW donkey α-goat IgG, IRDye 800CW goat α-rabbit.

### RNA sequencing and bioinformatic analyses

Total RNA was isolated from 5 × 10^6^ cells using RNeasy Mini Kit (Qiagen) and quantified using NanoDrop. Knockdown was assessed using qRT-PCR (Bio-Rad iScript One-Step RT-PCR Kit with SYBR Green on a MyiQ/iQ5 system) and western blots, then libraries for mRNA sequencing were constructed with unique adapters for each of three biological replicates. The samples were run on the same sequencing lane on an Illumina HiSeq 2000 with 50-cycle single-end reads. Data from the NKX2-2 mRNA sequencing experiment were uploaded to the NCBI Sequence Read Archive (SUB237370) and is freely available. Raw sequence reads were aligned with Novoalign (Novocraft Technologies Sdn Bhd) to an hg19 index. This index is supplemented with small sequences representing splice junctions within the transcript model derived from Ensembl76, which was created by the MakeTranscription application from the USeq suite (http://useq.sourceforge.net). SAM files resulting from this step were processed with SamTranscriptomeParser from the same suite and used as input for the RNA-seq analysis. Differential expression was determined by using USeq's RNA-seq wrapper for the Bioconductor package DESeq [66]. Heat map and gene set enrichment analyses were performed using R and GenePattern, respectively. Venn diagrams were generated using VennMaster (http://sysbio.uni-ulm.de/?Software:VennMaster). Functional annotation clustering analysis was done using the Database for Annotation, Visualization and Integrated Discovery (DAVID v6.7, http://david.abcc.ncifcrf.gov/home.jsp).

### Immunofluorescence studies

Staining of cells for immunofluorescence microscopy was performed using methods described previously [41], using the following reagents: normal goat serum (Santa Cruz sc-2043), mouse α-paxillin (BD Biosciences 610619), AlexaFluor-488 goat α-mouse IgG (Life Technologies A11029), AlexaFluor-568-phalloidin (Life Technologies A12380), DAPI (Life Technologies), Fluoromount-G (Southern Biotech 0100-01). Microscopy at 40X and 63X under oil immersion was performed using a Zeiss Axioskop 2 MOT Plus system, also previously described [41]. ImageJ was used to quantify cell area, while paxillin-positive focal adhesions were manually enumerated. Phalloidin-stained actin stress fibers were quantitated using an erosion decay method to generate a stress fiber thickness index (SFTI) as previously described [43, 44].

### Adhesion assay

3 × 10^5^ A673 cells from each condition were seeded in triplicate in 24-well plates and allowed to adhere for 2 h at 37°C and 5% CO _2_. Wells were gently washed with PBS, fixed with 3.7% formaldehyde for 15 minutes, and again washed with PBS. Adherent cells were stained with 1% Toluidine Blue for 1 h. Wells were washed with dH O and allowed to dry overnight. Cells were lysed and the dye solubilized with 2% SDS. Absorbance readings were done at 595 nm, using 2% SDS as blank.

### Migration assays

Scratch assays were performed as previously described [46]. Briefly, 2 × 10^6^ cells were seeded in triplicate into 6-well tissue culture plates, and allowed to reach confluency. A micropipette tip was used to make a wound on the monolayer across the vertical diameter of the well. Each day wells were washed gently with PBS and replaced with culture media containing 5% serum. Phase-contrast images were taken of 9 fields at 0, 24, 48, and 72 hours post-wounding using a 5X objective on a Zeiss Axiovert100 microscope and QCapture Pro 7 software. Wound area was quantified with ImageJ and data presented as percent wound area healed per day.

### Statistical analyses

Unpaired two-sample t-test analyses assuming unequal variance were performed in Microsoft Excel for qRT-PCR experiments and quantifications for cell area and focal adhesions. A log p rank test was performed for the Kaplan-Meier curve using GraphPad Prism 6.

## SUPPLEMENTARY MATERIAL, FIGURES AND TABLE


