# Supplementary material for: EWS/FLI utilizes NKX2-2 to repress mesenchymal features of Ewing sarcoma
Source: Genes Cancer. 2015 Mar;6(3-4):129–43. doi: 10.18632/genesandcancer.57 (PMC4426950; doi:10.18632/genesandcancer.57)
Supplement: Supplementary file 1 [file ganc-06-129-s001.pdf]

EWS/FLI utilizes NKX2-2 to repress mesenchymal features of ewing sarcoma

Supplementary Material

Table S1: List of NKX2-2 Repressed and Activated Gene Targets. We found that NKX2-2 repressed 189 and activated 49 genes at fold change ≥ 1.5 ($|\log_2 T/C| \geq 0.585$) and maximum DESeq FDR ≥ 50. "T/C" denotes the ratio of reads between "treatment" (NKX2-2 shRNA) and "control" (control shRNA). Genes in boldface are also EWS/FLI-regulated, i.e. genes that are in the Venn diagram intersections (Fig. 1c).

Repressed Genes			
**Ensembl ID**	**Gene Symbol**	**Max DESeq FDR**	**$\lvert Log_2(T/C)\rvert$**
ENSG00000175899	**A2M**	824.2042	2.5672
ENSG00000171201	SMR3B	839.0181	2.4857
ENSG00000130203	**APOE**	638.7277	2.2606
ENSG00000109846	**CRYAB**	750.5163	2.0917
ENSG00000113083	**LOX**	650.2720	2.0691
ENSG00000120708	**TGFBI**	447.5006	1.8439
ENSG00000182492	**BGN**	397.8915	1.7014
ENSG00000168542	**COL3A1**	388.0310	1.6943
ENSG00000100292	**HMOX1**	348.7940	1.6022
ENSG00000140092	**FBLN5**	287.2886	1.4978
ENSG00000106333	**PCOLCE**	277.2993	1.4858
ENSG00000164220	**F2RL2**	224.5895	1.3074
ENSG00000181104	**F2R**	197.7093	1.3003
ENSG00000163017	ACTG2	186.1635	1.2566
ENSG00000224729	**RP13-530H6.2**	198.2907	1.2327
ENSG00000166426	CRABP1	177.9007	1.2267
ENSG00000152377	**SPOCK1**	194.4080	1.2238
ENSG00000050165	**DKK3**	194.3323	1.2094
ENSG00000162998	FRZB	177.4267	1.1972
ENSG00000163661	**PTX3**	161.1237	1.1891
ENSG00000105270	**CLIP3**	173.1340	1.1832
ENSG00000122786	**CALD1**	197.7093	1.1795
ENSG00000174938	**SEZ6L2**	177.9007	1.1714
ENSG00000113721	**PDGFRB**	159.8752	1.1524
ENSG00000138080	**EMILIN1**	163.2299	1.1487

ENSG00000255887	HTR4	148.7123	1.1307
ENSG00000132718	**SYT11**	145.7101	1.1229
ENSG00000140682	**TGFB1I1**	178.7892	1.1204
ENSG00000001036	**FUCA2**	135.8940	1.1032
ENSG00000163297	**ANTXR2**	145.0749	1.0982
ENSG00000137558	PI15	135.0315	1.0911
ENSG00000084636	**COL16A1**	154.8618	1.0823
ENSG00000170445	**HARS**	116.6256	1.0579
ENSG00000140416	**TPM1**	162.9830	1.0578
ENSG00000159840	**ZYX**	146.2752	1.0574
ENSG00000147100	**SLC16A2**	129.2495	1.0493
ENSG00000163359	**COL6A3**	154.8618	1.0387
ENSG00000185585	**OLFML2A**	139.5245	1.0355
ENSG00000172020	**GAP43**	138.2877	1.0349
ENSG00000091129	**NRCAM**	131.3168	1.0223
ENSG00000163346	**PBXIP1**	118.8992	1.0092
ENSG00000130176	**CNN1**	157.1690	1.0060
ENSG00000175868	CALCB	97.6286	1.0018
ENSG00000007062	PROM1	119.5708	0.9975
ENSG00000143341	HMCN1	107.9254	0.9945
ENSG00000100095	SEZ6L	104.3363	0.9831
ENSG00000172638	**EFEMP2**	122.1316	0.9617
ENSG00000113140	**SPARC**	113.8953	0.9529
ENSG00000100968	NFATC4	95.6476	0.9523
ENSG00000148677	**ANKRD1**	144.9075	0.9509
ENSG00000174099	**MSRB3**	119.5708	0.9492
ENSG00000119630	**PGF**	112.9963	0.9484
ENSG00000180573	HIST1H2AC	95.3523	0.9442
ENSG00000142871	**CYR61**	122.6468	0.9414
ENSG00000141338	ABCA8	102.8714	0.9372
ENSG00000120896	**SORBS3**	107.5048	0.9319
ENSG00000117228	**GBP1**	98.6774	0.9256
ENSG00000115414	**FN1**	93.2659	0.9066
ENSG00000171195	MUC7	108.4639	0.9031
ENSG00000163395	**IGFN1**	89.1615	0.8949
ENSG00000174807	**CD248**	97.7315	0.8923

ENSG00000026297	**RNASET2**	84.3447	0.8848
ENSG00000112769	**LAMA4**	92.0180	0.8815
ENSG00000213694	**S1PR3**	93.4723	0.8723
ENSG00000164488	**DACT2**	91.3191	0.8670
ENSG00000123384	**LRP1**	94.8068	0.8653
ENSG00000123933	**MXD4**	92.1400	0.8647
ENSG00000109576	**AADAT**	88.8271	0.8565
ENSG00000232783	AC073135.3	86.1990	0.8549
ENSG00000132386	SERPINF1	79.2795	0.8540
ENSG00000103485	QPRT	80.5658	0.8507
ENSG00000211445	**GPX3**	95.0507	0.8500
ENSG00000136235	**GPNMB**	125.6224	0.8428
ENSG00000131724	**IL13RA1**	92.2947	0.8420
ENSG00000109323	**MANBA**	90.7411	0.8409
ENSG00000115718	PROC	81.4366	0.8408
ENSG00000168994	**C6orf145**	80.7035	0.8352
ENSG00000163485	**ADORA1**	92.1477	0.8341
ENSG00000164938	**TP53INP1**	79.2954	0.8321
ENSG00000171940	**ZNF217**	74.9894	0.8251
ENSG00000118733	OLFM3	74.4940	0.8250
ENSG00000126016	AMOT	79.1751	0.8215
ENSG00000144730	**IL17RD**	90.7412	0.8207
ENSG00000013016	**EHD3**	90.2576	0.8147
ENSG00000076706	**MCAM**	96.1121	0.8129
ENSG00000180998	GPR137C	62.2068	0.8104
ENSG00000133110	**POSTN**	93.8375	0.8089
ENSG00000164692	**COL1A2**	88.0884	0.8041
ENSG00000169231	**THBS3**	70.3029	0.8030
ENSG00000168280	**KIF5C**	67.2596	0.8029
ENSG00000155130	**MARCKS**	68.7284	0.8026
ENSG00000162490	**C1orf187**	88.9574	0.8025
ENSG00000168487	**BMP1**	78.8870	0.8007
ENSG00000119522	**DENND1A**	69.3145	0.8002
ENSG00000245532	**NEAT1**	61.6564	0.7993
ENSG00000168461	**RAB31**	77.1856	0.7985
ENSG00000176720	**BOK**	82.4016	0.7968

ENSG00000166750	**SLFN5**	66.4069	0.7968
ENSG00000170500	LONRF2	62.5051	0.7967
ENSG00000064042	LIMCH1	65.5743	0.7945
ENSG00000149212	SESN3	68.1630	0.7911
ENSG00000162493	PDPN	65.1762	0.7899
ENSG00000173698	GPR64	71.5143	0.7875
ENSG00000162804	**SNED1**	69.0734	0.7869
ENSG00000075223	**SEMA3C**	67.8865	0.7862
ENSG00000144455	**SUMF1**	65.5743	0.7844
ENSG00000169071	**ROR2**	75.8909	0.7837
ENSG00000256061	**DYX1C1-CCPG1**	68.4338	0.7803
ENSG00000134013	**LOXL2**	80.7819	0.7742
ENSG00000150593	PDCD4	63.3693	0.7726
ENSG00000166340	TPP1	80.7120	0.7698
ENSG00000007866	**TEAD3**	67.3072	0.7641
ENSG00000087303	**NID2**	67.6544	0.7616
ENSG00000166444	**ST5**	63.5447	0.7592
ENSG00000168016	TRANK1	72.5045	0.7586
ENSG00000090776	EFNB1	63.4265	0.7585
ENSG00000141756	**FKBP10**	68.4422	0.7580
ENSG00000138829	FBN2	58.2532	0.7577
ENSG00000168386	**FILIP1L**	69.6937	0.7573
ENSG00000179820	**MYADM**	65.5680	0.7567
ENSG00000135363	**LMO2**	57.9719	0.7542
ENSG00000137070	IL11RA	64.4975	0.7539
ENSG00000119655	**NPC2**	63.1146	0.7536
ENSG00000088882	**CPXM1**	66.3513	0.7523
ENSG00000197444	OGDHL	69.8468	0.7505
ENSG00000173548	**SNX33**	61.2146	0.7388
ENSG00000154262	ABCA6	62.7327	0.7380
ENSG00000107796	**ACTA2**	69.8468	0.7357
ENSG00000135406	PRPH	61.3089	0.7353
ENSG00000102032	RENBP	61.7957	0.7351
ENSG00000123200	ZC3H13	59.6177	0.7334
ENSG00000090238	YPEL3	60.3276	0.7327
ENSG00000154258	ABCA9	61.8086	0.7305

ENSG00000019549	**SNAI2**	72.7718	0.7304
ENSG00000132563	REEP2	59.5967	0.7296
ENSG00000229036	**RP1-20N2.6**	54.4579	0.7280
ENSG00000187714	SLC18A3	61.0677	0.7227
ENSG00000185924	**RTN4RL1**	57.4379	0.7204
ENSG00000115363	**FAM176A**	64.7503	0.7196
ENSG00000100196	**KDELR3**	62.9979	0.7189
ENSG00000129038	**LOXL1**	72.0864	0.7175
ENSG00000174233	**ADCY6**	56.6107	0.7171
ENSG00000173110	**HSPA6**	70.8810	0.7158
ENSG00000166147	**FBN1**	70.4821	0.7138
ENSG00000132205	**EMILIN2**	66.4069	0.7129
ENSG00000104325	DECR1	51.8177	0.7125
ENSG00000153208	MERTK	57.4382	0.7101
ENSG00000180596	HIST1H2BC	51.0862	0.7096
ENSG00000166033	**HTRA1**	66.6696	0.7074
ENSG00000073712	**FERMT2**	63.8742	0.7049
ENSG00000089169	RPH3A	58.0015	0.7037
ENSG00000103710	RASL12	52.0173	0.7024
ENSG00000163430	**FSTL1**	54.7960	0.6943
ENSG00000184500	**PROS1**	52.8782	0.6936
ENSG00000254087	**LYN**	53.2529	0.6934
ENSG00000180139	**RP11-399O19.5**	59.4507	0.6920
ENSG00000087245	**MMP2**	74.3663	0.6888
ENSG00000144668	**ITGA9**	55.5170	0.6885
ENSG00000137507	**LRRC32**	64.4975	0.6875
ENSG00000135424	ITGA7	54.2254	0.6854
ENSG00000035862	**TIMP2**	59.4507	0.6841
ENSG00000145703	IQGAP2	55.9574	0.6839
ENSG00000148926	**ADM**	54.9067	0.6836
ENSG00000136153	**LMO7**	69.2007	0.6835
ENSG00000075213	**SEMA3A**	53.0944	0.6815
ENSG00000024422	**EHD2**	70.2261	0.6797
ENSG00000146904	**EPHA1**	52.8476	0.6784
ENSG00000064666	**CNN2**	51.0883	0.6764
ENSG00000100097	**LGALS1**	56.0601	0.6757

ENSG00000123989	**CHPF**	59.5704	0.6753
ENSG00000109790	**KLHL5**	50.8233	0.6749
ENSG00000124749	COL21A1	51.7971	0.6745
ENSG00000168140	**VASN**	55.5914	0.6706
ENSG00000134531	**EMP1**	51.4953	0.6703
ENSG00000197712	**FAM114A1**	74.0854	0.6691
ENSG00000170558	**CDH2**	55.2606	0.6657
ENSG00000136859	**ANGPTL2**	64.4887	0.6649
ENSG00000145248	**SLC10A4**	54.7960	0.6649
ENSG00000179954	**SSC5D**	55.6596	0.6588
ENSG00000176046	**NUPR1**	54.9144	0.6544
ENSG00000064309	**CDON**	50.7290	0.6529
ENSG00000106366	**SERPINE1**	56.9858	0.6427
ENSG00000131981	**LGALS3**	52.5960	0.6402
ENSG00000011028	**MRC2**	56.4280	0.6401
ENSG00000158089	**GALNT14**	50.5565	0.6331
ENSG00000118523	**CTGF**	52.6271	0.6294
ENSG00000130635	**COL5A1**	52.9122	0.6276
ENSG00000163909	**HEYL**	51.0532	0.6096
ENSG00000149591	**TAGLN**	50.0557	0.5875
**Activated Genes**			
**Ensembl ID**	**Gene Symbol**	**Max DESeq FDR**	**$\|Log_2(T/C)\|$**
ENSG00000128245	YWHAH	221.7330	1.4394
ENSG00000158710	TAGLN2	157.9439	1.2271
ENSG00000064300	**NGFR**	146.8393	1.1719
ENSG00000049540	ELN	127.8538	1.1311
ENSG00000140497	SCAMP2	117.3317	1.0755
ENSG00000137309	HMGA1	108.8962	1.0253
ENSG00000166803	**KIAA0101**	104.7058	0.9707
ENSG00000009709	**PAX7**	86.6807	0.9616
ENSG00000101187	SLCO4A1	89.0895	0.9250
ENSG00000079215	**SLC1A3**	86.1420	0.9049
ENSG00000112759	**SLC29A1**	81.4337	0.8922
ENSG00000186867	**QRFPR**	87.2617	0.8907
ENSG00000135447	**PPP1R1A**	81.5021	0.8899

ENSG00000158195	WASF2	78.8870	0.8809
ENSG00000169894	**MUC3A**	84.3078	0.8776
ENSG00000124164	VAPB	77.2833	0.8664
ENSG00000085552	**IGSF9**	80.7035	0.8663
ENSG00000138061	CYP1B1	81.5021	0.8430
ENSG00000159167	STC1	73.5211	0.8410
ENSG00000117289	**TXNIP**	67.0722	0.8307
ENSG00000071655	MBD3	66.4069	0.8216
ENSG00000249906	**RP5-1029K10.2**	70.9345	0.8201
ENSG00000187867	**PALM3**	68.1630	0.7994
ENSG00000213390	**ARHGAP19**	66.9043	0.7947
ENSG00000081181	**ARG2**	66.3513	0.7900
ENSG00000166342	**NETO1**	66.8842	0.7828
ENSG00000173805	**HAP1**	65.5680	0.7654
ENSG00000198576	**ARC**	61.3460	0.7649
ENSG00000166974	MAPRE2	63.4265	0.7561
ENSG00000080819	CPOX	58.0015	0.7519
ENSG00000173890	**GPR160**	53.8457	0.7515
ENSG00000126785	RHOJ	54.9563	0.7467
ENSG00000172757	CFL1	53.0042	0.7441
ENSG00000054611	TBC1D22A	50.5441	0.7417
ENSG00000131238	PPT1	53.0363	0.7396
ENSG00000135446	CDK4	55.7100	0.7380
ENSG00000057294	**PKP2**	55.8437	0.7289
ENSG00000149269	PAK1	55.1639	0.7271
ENSG00000172236	**TPSAB1**	54.3280	0.7254
ENSG00000171766	**GATM**	54.9487	0.7203
ENSG00000072736	**NFATC3**	52.5705	0.7202
ENSG00000128594	**LRRC4**	52.9122	0.7202
ENSG00000129484	**PARP2**	53.2527	0.7194
ENSG00000168765	**GSTM4**	50.8257	0.7172
ENSG00000181744	C3orf58	54.6557	0.7170
ENSG00000113368	**LMNB1**	51.6650	0.7152
ENSG00000165495	**PKNOX2**	52.9258	0.7141
ENSG00000164362	**TERT**	51.2488	0.7124
ENSG00000165731	**RET**	52.0706	0.7109

FIGURE S1

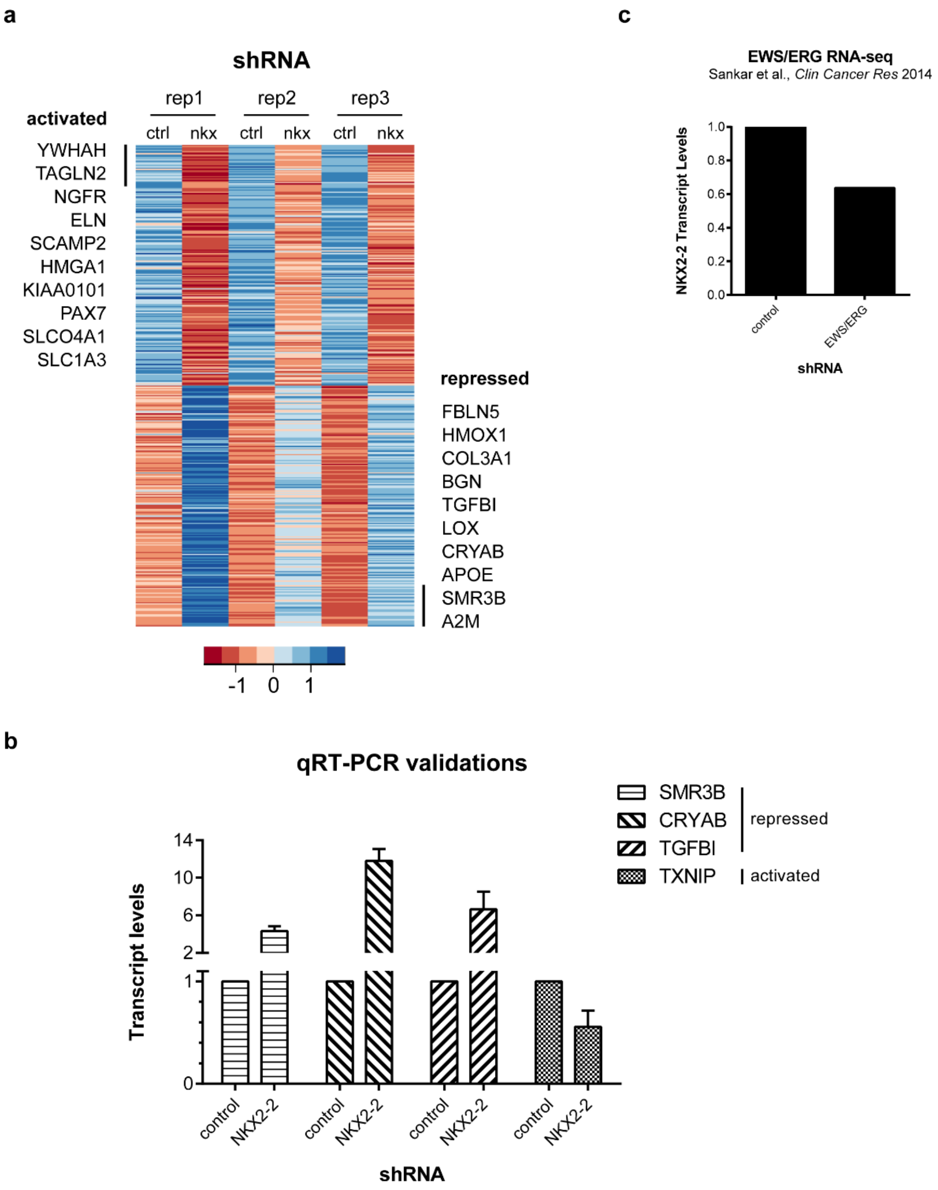

Figure S1: Key NKX2-2-regulated genes. (a) Heat map shows top 200 activated or repressed genes of NKX2-2 in A673 cells. The control shRNA and NKX2-2 shRNA lanes for each of three replicates are indicated. The top 10 genes in each set are enumerated. (b) qRT-PCR validation of select NKX2-2 target genes. (c) NKX2-2 is an activated target of EWS/ERG in TTC466 cells.

FIGURE S2

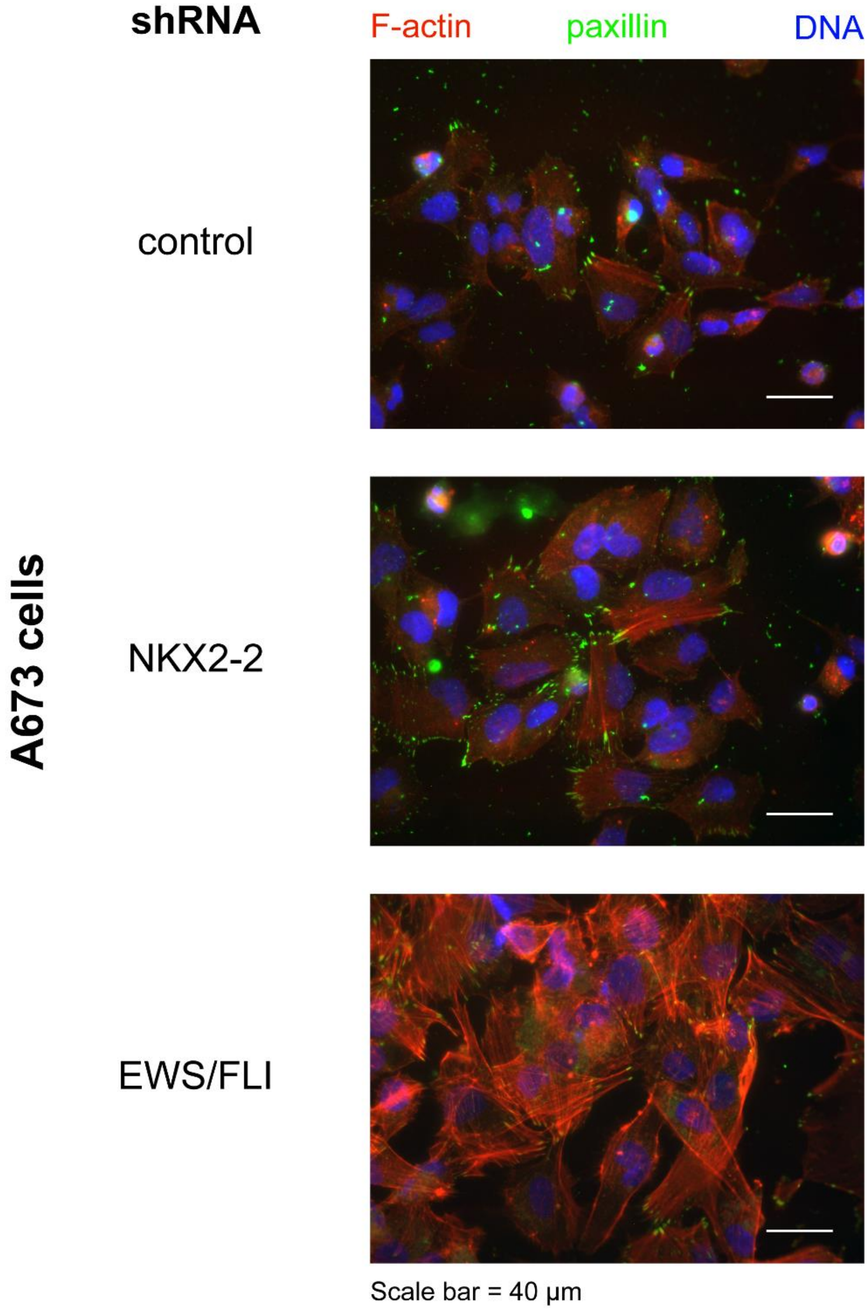

Figure S2: Immunofluorescence 40X fields of A673 cells harboring control, NKX2-2, or EWS/FLI shRNA. Merged images of IF staining for F-actin (red), paxillin (green), and DNA (blue) are shown.

FIGURE S3

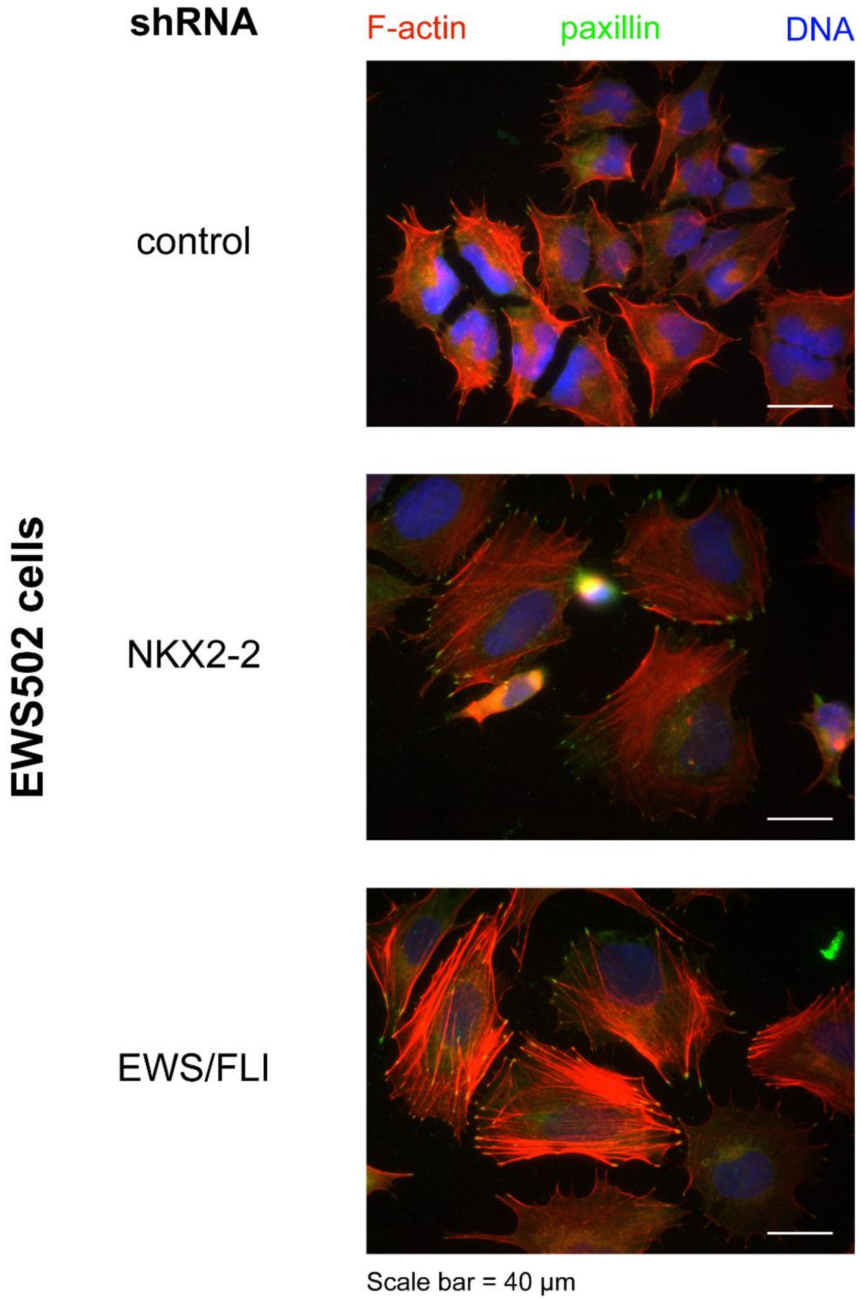

Figure S3: Immunofluorescence 40X fields of EWS502 cells harboring control, NKX2-2, or EWS/FLI shRNA. Merged images of IF staining for F-actin (red), paxillin (green), and DNA (blue) are shown.

FIGURE S4

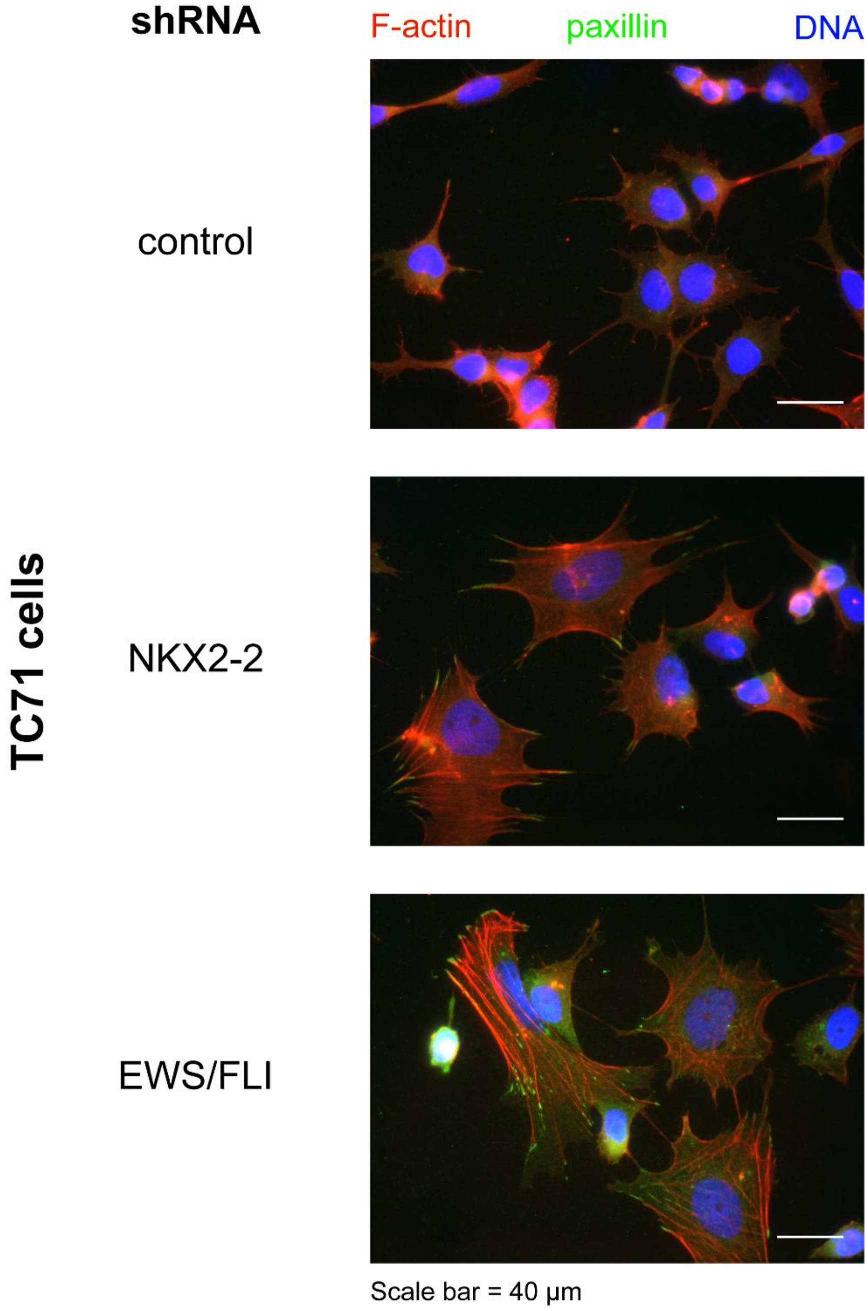

Figure S4: Immunofluorescence 40X fields of TC71 cells harboring control, NKX2-2, or EWS/FLI shRNA. Merged images of IF staining for F-actin (red), paxillin (green), and DNA (blue) are shown.

FIGURE S5

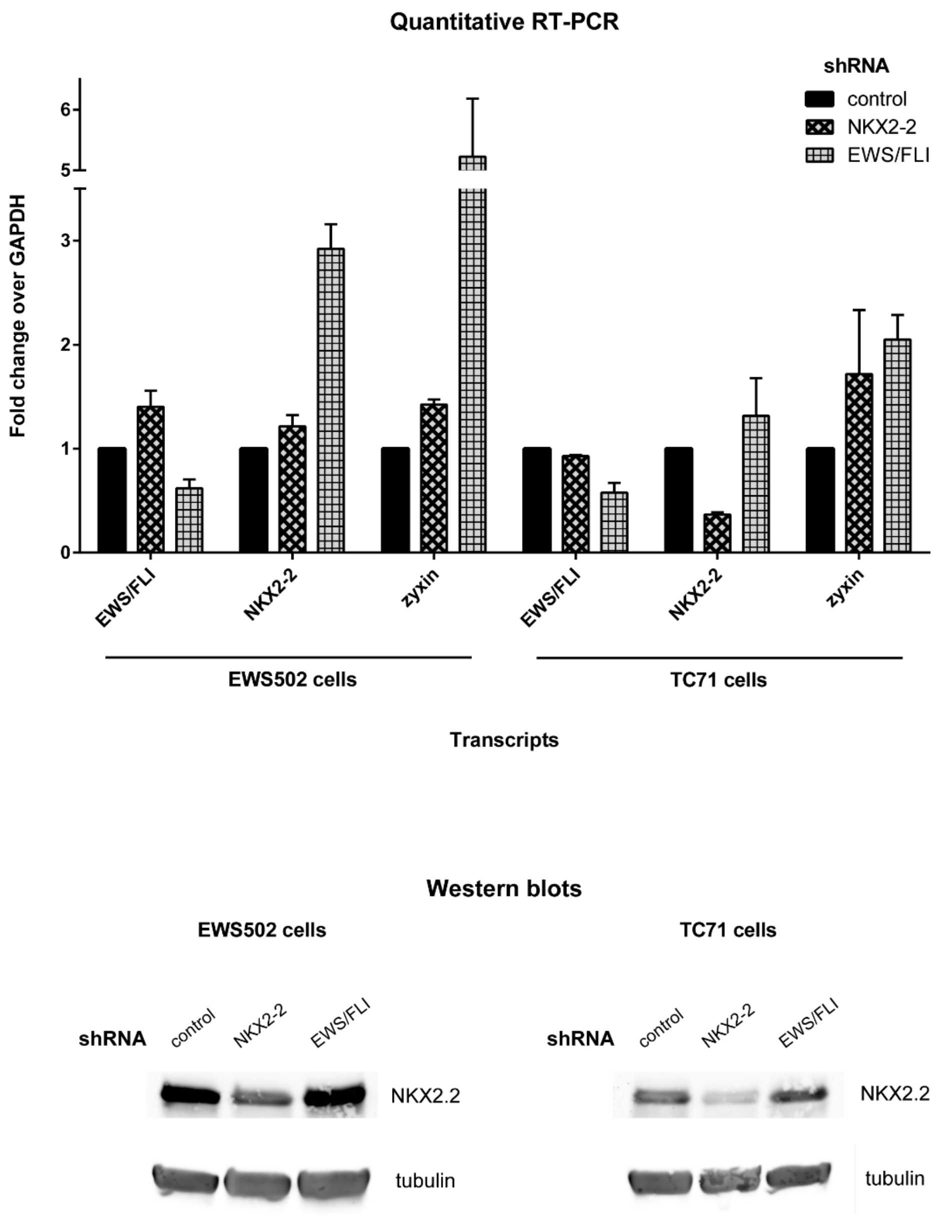

Figure S5: qRT-PCR and western blots of EWS502 and TC71 cells. *Top*, qRT-PCR results using EWS/FLI-, NKX2-2-, and zyxin-specific primer sets. Shown are fold-change values, normalized against GAPDH levels, for cells harboring control, NKX2-2 or EWS/FLI shRNA for either cell line. *Bottom*, NKX2.2 and tubulin protein levels for each knockdown condition in either cell line.

FIGURE S6

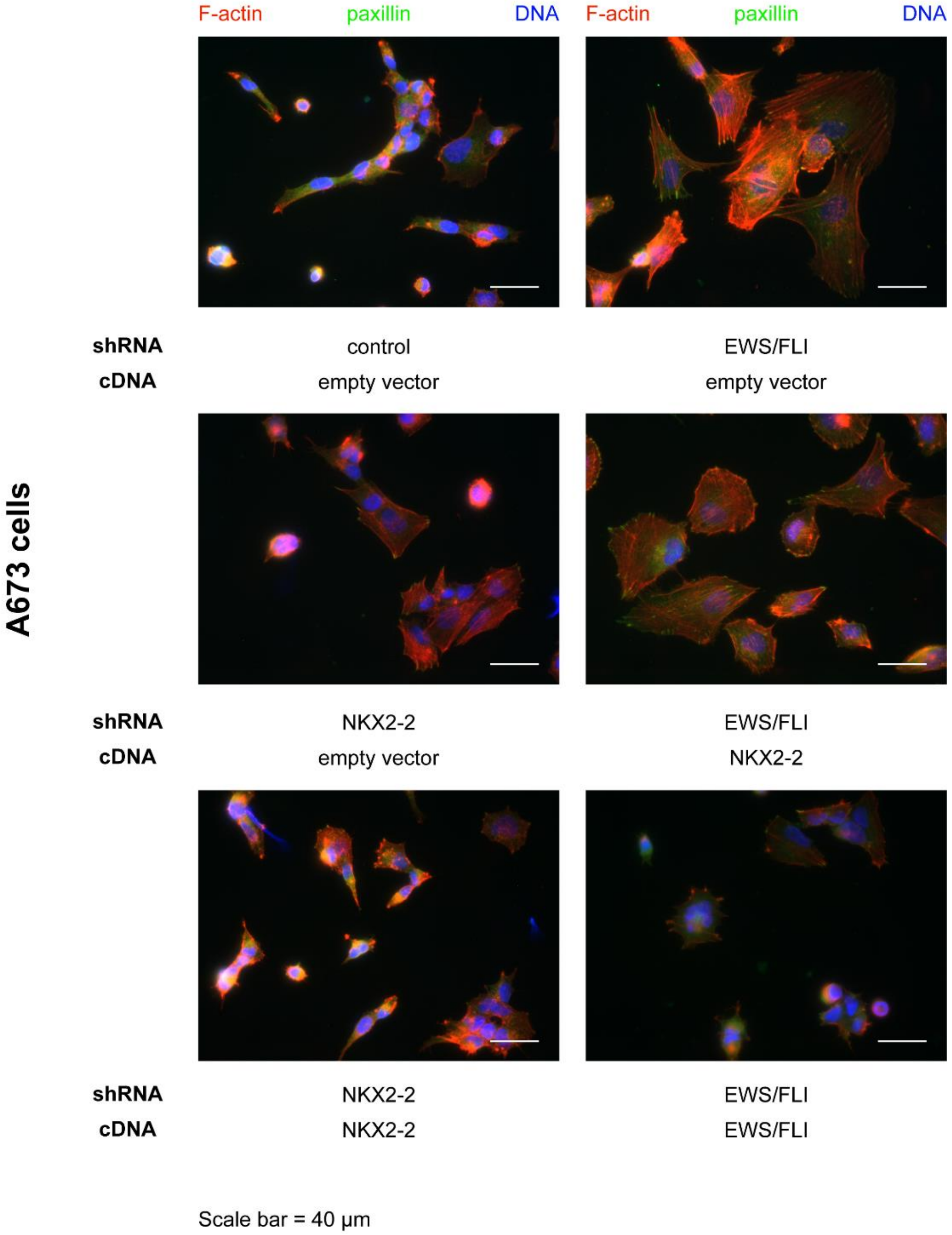

Figure S6: Immunofluorescence 40X fields of A673 knockdown-rescue cells. Cells harboring control, NKX2-2, or EWS/FLI shRNA, and empty vector (pMSCV-hygro) or cDNA for 3XFLAG::NKX2-2 or 3XFLAG::EWS/FLI, as indicated. Merged images of IF staining for F-actin (red), paxillin (green), and DNA (blue) are shown.
